# Raw Materials, Technology, Healthcare Applications, Patent Repository and Clinical Trials on 4D Printing Technology: An Updated Review

**DOI:** 10.3390/pharmaceutics15010116

**Published:** 2022-12-29

**Authors:** Mohammad Sameer Khan, Sauban Ahmed Khan, Shaheen Shabbir, Md Umar, Sradhanjali Mohapatra, Tahir Khuroo, Punnoth Poonkuzhi Naseef, Mohamed Saheer Kuruniyan, Zeenat Iqbal, Mohd Aamir Mirza

**Affiliations:** 1 School of Pharmaceutical Education and Research, Jamia Hamdard University, New Delhi 110062, India; 2 Department of Pharmaceutics, School of Pharmaceutical Education and Research, Jamia Hamdard University, New Delhi 110062, India; 3 PGx Global Foundation, 5600 SOUTH Willow Dr Ste 101, Houston, TX 77840, USA; 4 Department of Pharmaceutics, Moulana College of Pharmacy, Perinthalmanna 679321, India; 5 Department of Dental Technology, College of Applied Medical Sciences, King Khalid University, Abha 61421, Saudi Arabia

**Keywords:** shape memory polymer, 4D printing, medical devices, additive manufacturing, health care

## Abstract

After the successful commercial exploitation of 3D printing technology, the advanced version of additive manufacturing, i.e., 4D printing, has been a new buzz in the technology-driven industries since 2013. It is a judicious combination of 3D printing technologies and smart materials (stimuli responsive), where time is the fourth dimension. Materials such as liquid crystal elastomer (LCE), shape memory polymers, alloys and composites exhibiting properties such as self–assembling and self-healing are used in the development/manufacturing of these products, which respond to external stimuli such as solvent, temperature, light, etc. The technologies being used are direct ink writing (DIW), fused filament fabrication (FFF), etc. It offers several advantages over 3D printing and has been exploited in different sectors such as healthcare, textiles, etc. Some remarkable applications of 4D printing technology in healthcare are self-adjusting stents, artificial muscle and drug delivery applications. Potential of applications call for further research into more responsive materials and technologies in this field. The given review is an attempt to collate all the information pertaining to techniques employed, raw materials, applications, clinical trials, recent patents and publications specific to healthcare products. The technology has also been evaluated in terms of regulatory perspectives. The data garnered is expected to make a strong contribution to the field of technology for human welfare and healthcare.

## 1. Introduction

**Figure 1:**
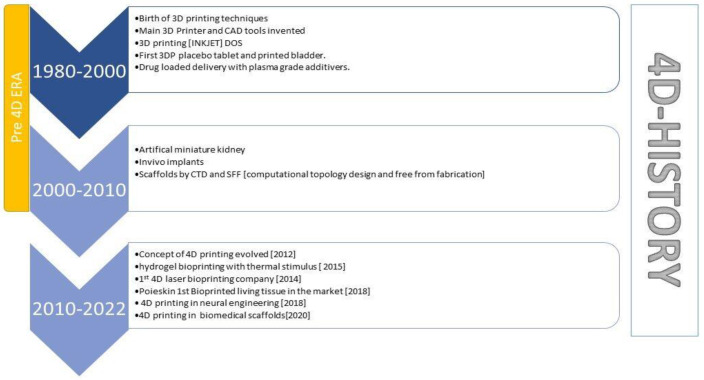
The timeline briefly explains the evolution of 4D printing technology. The timeline is broadly divided into two eras, pre 4D era, which highlights the invention of 3D printing technology. The pre-4D era informs us of the major contribution of 3D printing technology in the healthcare sector and the components and tools used in 3D. The second era is marked by the evolution of 4D printing in 2012, the discovery of various hydrogels and the application of 4D in various sectors.

The introduction of the fourth dimension (i.e., time) in the field of science resulted in the invention of 4D printing (4DP) technology. The previously successful additive manufacturing, i.e., 3D printing (3DP), laid down the foundation of 4DP. The new development is an amalgamation of 3DP and time. Tibbit proposed the concept of 4DP [1], which has since been defined as 3D-printed structures that exhibit targeted shape or property transformation under external stimulus, exhibiting the shape memory effect (SME). The SME includes two basic steps, firstly programming, followed by recovery step. The 3DP process in conjugation with smart materials (materials having special properties which can vary their shape and structure, once activated by an external supply of force) is used in 4DP to construct dynamic structures. 4D includes the use of various smart materials such as smart polymers, alloys, etc. Hydrogels in smart material polymer are a popular choice for raw material and a few smart material alloys have also caught the researcher’s interest. There are different methods of 4DP such as stereolithography, fused deposition method (FDM), etc. The stereolithography method is based on layer-by-layer printing and controlling factors such as scanning speed, coverage time and use of UV laser. FDM is based on the shape memory effect and speed, temperature, etc., are factors that influence the process. Figure 1 depicts a timeline explaining the evolution of 4DP technology. This timeline briefly explains the evolution of 4DP Technology. It is broadly divided into two eras pre-4D era, which highlights the previous invention of 3DP technology. The pre-era informs us of the major contribution of 3DP in the healthcare sector and the components and tools used in 3DP. The second era is marked by the evolution of 4DP in 2012, the discovery of various hydrogels and the application of 4DP in various sectors.

In response to any stimulus, 4D-printed objects change their form or functionality. Liquid, UV light, pressure, pH exposure to the magnetic field, etc. are a few examples of such stimuli [2]. Under the influence of environmental incitements such as temperature, light, or humidity, 4DP has led to the change of the printed 3D structure into a new and more complicated shape. The advantages of 4DP can be listed as multilateral designing, extra programmed functionality, preservation of raw materials and reduced production time, which is further backed by mathematical modelling [3]. With the application of 4DP, an article may be programmed to change shape as it comes into contact with its environment. Packaging, medical, construction and automotive are just a few of the industries that rely heavily on 4DP. The industries where 4DP is most expected to be helpful include healthcare, automobile, textile, architecture, aerospace, military, defense, etc. [2]. In the healthcare sector, 4DP has proved to be a boon by making it possible to create self-adjusting stents and artificial muscle, and has a crucial role in the drug delivery system. Customized and more adjustable, biocompatible devices such as stents, and scaffolds have better outcomes, even in live-saving circumstances [3,4]. Further, these structures have shape-changing, self-healing abilities, and a propensity for self-assembly [5].

Ding et al. suggested an alternate method in which a temporary shape is first produced using a Stratasys multi-material J750 printer, followed by the use of heat stimuli to form a permanent shape [6]. The degree of change manifested by the structure determines the difference between the 4DP of a single material and of several materials. The degree of alteration in a single material 4DP process depends on the smart materials’ response to a quantitative stimulus. How quickly a single material component changes configuration after activation depends on the degree of change. Multi-material components printed in four dimensions are evaluated for alterations, particularly in terms of individual form and structural modification. The design of the activation mode, folding, bending and other motions may all be used to calculate task-oriented actuation. According to a market research study, the value of the 4DP market was $63.00 million in 2019 and will be $555.60 million by 2025 [7]. Although this is such a fledgling field, its advancement in pharmaceuticals is still confined to laboratory research rather than the commercial market.

This review elucidates 4DP technology with special reference to additive manufacturing and includes a discussion about raw materials and stimuli involved in this technology. Further, it contains advancement in 4DP and how it is improved over 3DP along with its applications in health care. Additionally, it enlists data related to 4DP in the form of patents and clinical trials. Searches for research publications published up to 2022 are made using keywords like “4D printing” and “4D printing in medicine,” and then a bibliometric analysis of these papers was carried out. For use in the medical/pharmaceutical industry, additional pertinent publications were investigated. The technology has also been evaluated in terms of regulatory perspectives. The data collected in this article is expected to make a strong contribution to the technology of human welfare and healthcare.

## 2. Raw Materials

4DP makes use of raw materials such as alloys, ceramics and polymers that respond to stimuli. These stimuli may be temperature, pH, moisture, etc. The emphasis in 4DP is primarily on the deformation mechanism, geometry [8], stress distribution, pattern design [9] and recovery sequence [10]. Hence, the raw materials display SME, which means they can revert to their original shape and structure. These materials have self-assembling, self-healing and self-adjusting properties. One major classification of 4DP smart materials is based on thermal responsiveness: shape memory effect (SME) [11] or shape change effect (SMC) [12]. SME materials are further categorized into Shape memory alloys (SMA), shape memory polymers (SMPs), shape memory gel, shape memory ceramics, and shape Memory Composite (SMC). Further, SMPs are made up of a monomer (soft component) and a crosslinker (hard component). These are a wide class of polymers used in 4DP, such as stimuli-responsive SMP, SMP-based composite, etc. Stimuli-responsive hydrogel, such as water responsive, thermal-responsive, etc. is commonly used for this. The SMC is made by combining SMP and SMA to overcome their respective drawbacks. Furthermore, the SMP is preferred over SMA as they are lighter, more flexible and exhibit more biocompatibility than SMA. Additionally, they can withstand a large magnitude of stress and have less energy consumption. The SMPs are easy to print and have a large glass transition temperature (Tg) range, generally greater than the operating temperatures. They can be programmed to recover their original shape when cooled at no loading under certain mechanical treatments and specific heat above Tg [13]. 4D printing (via selective design) aids in optimizing shape recovery (at lower temperatures and faster speeds) while focusing on a specific task such as gripping [14].

The SMPs include hydrogels that can change their volume in response to stimuli [15]. These are hydrophilic in nature, showing swelling and de-swelling behaviour. These include moisture-responsive hydrogels such as PEG (Polyethylene glycol), thermo-responsive hydrogels such as PNIPAm (Poly N-isopropyl acrylamide) and chemo-responsive hydrogels such as poly acrylic acid. PNIPAm is the most widely studied thermo-responsive polymer that decreases in volume at a temperature above LCST (Lower Critical Solution Temperature) as the network of the bond collapses. The LCST is 32 °C. Due to the so-called “coil-globule transition,” which occurs when polymer chains in the solvent collapse or expand at certain temperatures, the temperature-sensitive hydrogels experience reversible volume change. One of the most commonly researched thermo-responsive materials is PNIPAm. In SMP, other components such as alginate are also incorporated with SMPs to improve their properties and functioning. The mechanism of their chemical-stimuli response is attributable to polymer chain crosslinking and dissociation when interacting with ions. Because the polymer chains in chemically responsive polymers are primarily linked by electrostatic interactions, changes in ion concentration will interfere with the strength of the electrostatic interaction and affect the properties of the hydrogels. For example, alginate is added to PNIPAm. The hydrogel’s response to stimuli is through cross-linking along with the dissociation of polymer chains [16]. The pH-sensitive materials have different functional groups imparting a weak basic or acidic character. Another important topic is multi stimuli-responsive hydrogels, which have a high potential for multifunctional applications. Ma et al. developed a temperature and pH-responsive perylene bisimide-functionalized hyperbranched polyethyleneimine (PBI-HPEI)-graphene oxide PNIPAm (GO-PNIPAm) hydrogel [17,18].

The SMAs are metal alloys existing in multiple phases or states showcasing super-elastic behaviour [19]. SMA has high yield strength and Young’s modulus of elasticity. nitinol, iron-based alloys such as Fe-Mn-Si alloy are a few examples of SMA. The SMA nitinol undergoes transformation of phase is dependent on the transition of austenite and martensite crystalline phases. The phase transition is in response to temperature.

The active material used in 4DP is not limited to single materials such as SMPs or hydrogels. When observed over time, nonactive and multi-material structures of suitable composition can also exhibit shape-changing effects. Polymer carbon nanotube (PCN) composites are formed when carbon nanotubes are added to polymeric materials in extremely small amounts. These nanotubes impart distinct mechanical and electrical properties to the PCN, permitting the material to use various shape-changing actuation patterns. This can be used to create a variety of PCNs, each of which can exhibit distinct stimuli–responsive behaviour and thus be used for a wide range of purposes, including those in the health sector. Polymers mixed with Iron Oxide nanoparticles and carbon nanotubes were used to create a wide range of shapes [20,21]. This is accomplished by introducing heat at various radio frequencies. Ceramics, in general, are extremely brittle and thus fail easily at small strains. Certain ceramics with a fine structure and few grain junctions, on the other hand, can withstand higher magnitudes of stress without cracking and thus have the potential to serve as smart materials. These compounds’ high strength and refractory nature make them ideal for temperature-activated shape memory applications. The addition of graphene nanocomposites to thermally activated SMPs increased the composite’s strength and allowed for greater deformation without fiber breakage. The addition of graphene reduces the crosslinking density of the SMP, resulting in an extremely tough composite. Because of their favourable mechanical properties, excellent electrical and thermal conductivities and low cost, graphene-based compounds have also been investigated as potential smart materials. Graphene-based smart materials have been used to manufacture photovoltaic devices, sensors, actuators and drug delivery biomedical devices due to their excellent properties. Organic materials, such as plant oil, have also been used to create shape memory materials. This material has improved mechanical properties and has the potential to be used as a biodegradable smart material [1,22].

Table 1 illustrates raw materials used in 4DP with their applications and properties. These raw materials include SMPs, SMAs and magnetic nanoparticles having specific stimuli such as temperature, pH, magnetic field, etc., with their characteristic properties. SMPs may be used as copolymers and SMAs generally respond to temperature. Further, several applications are enlisted including their use in drug delivery systems, biomedical devices and smart muscles.

### 2.1. Composites-Based Smart Materials

Shape memory polymer-based composites, hydrogels and fiber-reinforced composites are popular for changing shape and other properties in response to water and heat. Structures can also be altered using controlled buckling, pneumatic transformation, stress-induced curing and thermally controlled swelling. [23]. It has been claimed that printing shape memory polymer smart material, that deforms when exposed to temperature variations, like the structure of a plant leaf, improves the structural and mechanical qualities of blades [24]. The hydrogel-based composite contracts and expands as the temperature rises from 25 to 40 °C and eventually to 65 °C [25]. Some examples of composite-based smart materials used in 4DP technology include piezoelectric, electrostriction, thermoelectric and shape memory alloys.

### 2.2. Liquid Crystal Elastomers

Liquid crystal elastomers (LCE) are an additional type of stimulus-responsive smart materials having a reversible and anisotropic shape-changing capability as a result of stimuli such as light, heat, magnetic field and electric field, etc. [26,27,28]. When the temperature exceeds or falls below nematic-isotropic temperature (*T**_NI_***), LCEs undergo macroscopic contraction or elongation. In most cases, cross-linking liquid crystals in mesogen units produce LCE. This can be prepared more easily and is capable of rapid and intricate deformation due to the cross-linking and alignment of the mesogen units [29]. These are the materials for which these deformations are found to be most suited for a variety of applications, including artificial tissue, implants and sensor [30,31,32]. In order to prepare liquid crystal elastomers (LCEs) molecules for alignment, some researchers employed shear pressures produced by direct-writing printing and quick photo-cross-linking to produce negative coefficients of thermal expansion or snap-through deformations [33].

### 2.3. Multifunctional Materials

Multifunctional materials exhibit variations in several functional characteristics such as electrical and thermal conductivity, optical, self-healing and aesthetic properties, that vary with form, shape, size, and dimension change [34]. Therefore, it has been determined that such materials are most suited for simultaneous modification in shape and functional property. Moreover, 4DP of multifunctional materials is utilized in 4D organ-printing, knee and hip replacement and other biomaterial replacements [35].

To develop multifunctional meta-materials and to conduct uniaxial tension testing, a soft polymeric matrix supplemented with micro-structured support was used [36]. In addition to biological applications, the inclusion of more than two elements increases the stiffness of material under loading conditions. The improvement of numerous functional features of multifunctional materials makes them suitable for a wide range of bio-printing applications, such as 4D-printed tissues and tailored medical devices [37,38,39].

### 2.4. Single and Composite SMPs

The primary benefits of SMPs over other smart materials can be described as their substantial recovery capacity, low cost, lightweight, ease of processing and high shape deformability [40]. In response to various stimuli (light, heat, moisture, electric field, magnetic field, etc.), these materials have the proficiency to recover from substantial deformations. The Tg of SMPs determines the deformability of the shape memory polymers. When the Tg is achieved, the polymer begins to move and gradually becomes flexible. After this, the force applied causes deformation in the shape of the material and creates a new configuration. Subsequently, the material is refrigerated to obtain a new form. At any point in the process, the required degree of deformation can be accomplished by first heating the material to a temperature higher than the transition temperature, then adding load and cooling once the load has been removed [41].

SMPs have several benefits over inorganic ceramics and metallic smart materials such as low density, chemical stability, ease of manufacture, increased stress tolerance and improved strain recovery [42]. Due to their key properties, namely biodegradability, biocompatibility, and dynamic decomposition rate, SMPs are of significant importance in biomedical applications like drug delivery systems [43]. Relative low melting points and economical production of these materials have promoted their usage in additive manufacturing methods [44].

## 3. 4DP Technology

4DP is an evolution of 3DP where additive manufacturing printing techniques are employed. In other words, adapting 3DP processes for 4DP of SMP or smart materials requires minor adjustments. In order to produce the desired shape-changing materials as per estimation or for optimal application, an air circulation system may be incorporated into 3DP’s traditional FDM technology. This would cool the SMP below its Tg and, after these small alterations, previous 3DP methods such as SLA, digital laser writing and inkjet printing can also be employed. This section will give details about the several printing processes utilized in 4DP along with the stimuli that are responsible for shape transformation.

### 3.1. Additive Manufacturing (AM)

The process of layer-by-layer printing of a computer-aided design (CAD) model is known as AM [45]. This process involves significantly less mechanistic involvement and yields higher levels of precision. Frequently, additive manufacturing is juxtaposed with computer numerical control (CNC) machining, which is a material removal method, otherwise known as a subtractive manufacturing process. Therefore, the subtractive approach generates a significant amount of waste materials. On the contrary, AM is more design-flexible, cost-effective and environmentally benign. The fabrication method of 4DP is identical to that of 3DP using CAD software, employing the initial step of AM to create a CAD design of the desired object [46]. The file is then saved using the standard tessellation language file extension (STL file). Before the object is produced, stages such as proper settings, landscape, portrait printing, cartridge filling and thickness selection must be completed. The following sections depict the various layering techniques.

#### 3.1.1. Digital Light Processing (DLP)

Digital light processing (DLP) is based on the technique of vat polymerization [47]. In this procedure, a vat of liquid resin is polymerized or cured under a light source [48]. Under UV light provided by a light curing unit (LCU) or laser source, the drop of resin is cured. The laser then converts the liquid into a solid substance, layer by layer, forming the product.

#### 3.1.2. Direct Ink Writing (DIW)

The process of regulating the orientation of an anisotropic filler inside a polymer matrix is required for direct ink writing. Because of this, tension is generated, which is then successively modulated via ink writing for each pixel. In the biomedical area, DIW printers have been used to print high-strength biodegradable scaffolds and for tissue engineering. Printing extremely flexible and self-healing shape memory elastomers were accomplished through the use of a UV-assisted DIW technique. Because of the self-healing capabilities of these elastomers, they could be potentially employed as components in biomedical repair systems.

#### 3.1.3. Digital Laser Writing (DLW)

DLW is employed to create samples with dimensions beyond a few microns and at the submicron range. This approach is appropriate for manufacturing at the microscopic level and enables the 3DP of complex and intricate shapes with accurate dimensions at the scale of micrometers to nanometers [49]. The DLW polymerization method is designed on the basis of the polymerization threshold model, where resins are transparent to the near-infrared spectrum and the spontaneous absorption of two or more photons results in the formation of the desired materials. Self-healing shape memory polymers that are extremely extensible have been printed using the UV-assisted DLW process. Moreover, biodegradable scaffolds with great strength have been created using this method. The technique has wide applications in tissue engineering [50].

#### 3.1.4. Direct and Binder 3DP

Both direct 3DP and binder 3DP utilize the same inkjet printing technology. In direct 3DP, polymer and wax are utilized in place of ink and the liquid material is released through the up-and-down movement of the nozzle. These liquid polymers and waxes quickly solidify and form a layer of solid material. Rapid prototyping and multi-jet modelling are also advantages of this type of additive manufacturing (MJM) [51]

In binder-based 3DP, the printer extrudes two distinct substances, a fine powder and a liquid binder [52]. Each layer is composed of a combination of these two distinct substances. Consequently, one of the primary benefits of binder 3DP is that different materials can be selected throughout the manufacturing of the same product design. Furthermore, thick and porous graphene-based devices were created in research using direct and binder 3DP technologies. The device was capable of retaining 80% of its capability [53].

#### 3.1.5. Selective Laser Sintering (SLS)

SLS requires the melting and sintering of plastics and metals [54]. In this process, the raw material is first heated with a laser to a temperature just below its melting point so that it could be melted, and then the molten substance is solidified [55]. In case of metal, however, melting can be used instead of sintering to produce the end product owing to the possibility of minimal porosity and voids during melting. Generally, it is used to print hearing aid implants, thus successfully establishing its application in printing medical devices.

#### 3.1.6. Stereolithography Apparatus (SLA)

SLA is based on the vat photopolymerization strategy which uses a laser beam and a vat of liquid plastic photopolymers [56]. By using a laser beam, a layer of photopolymer is hardened and then, to create the final result, a layer-by-layer process is conducted. The basic components of SLA include a tank of liquid photopolymer resin or plastic, a high-powered UV laser, a platform and a computer interface for controlling the thickness and movement of the interface and laser. The SLA 3DP technique can be divided into several steps: (1) A CAD. stl file, which must be prepared for the component design, then this stl [unit] file is imported into slicer software, which will convert it into G-code for the machine’s required movement instructions; (2) When the procedure starts, the laser strikes the photosensitive resin, solidifying the liquid. Here, a computer-controlled mirror directs the laser to the specified coordinates; (3) After all of the layers have been applied, the model is removed from the platform and cured in a UV oven. This post-print curing helps the objects to achieve maximum strength and become more stable [57].

One of the key features of SLA is its capacity to generate high-resolution objects of varying sizes; objects ranging in size from submicron to decimeters can be made by using this technique. While the majority of AM (Additive manufacturing) methods are capable of producing structural features on the scale of 50–200 microns [58], the precision of SLA-printed SMPs is between 0.1 mm–1 μm [59].

#### 3.1.7. Fused Filament Fabrication (FFF)

FFF is also known as fusion deposition modelling or melt material extrusion (MME). It is the most affordable and widely accessible 3DP and 4DP technology [60]. Here, a spool of filament is inserted into the 3D printer and feed is supplied through an extrusion head nozzle in the material extrusion equipment. Then by using a pump, the filament goes to the heated nozzle where the desired temperature of the nozzle melts the filament. The extrusion head moves in predetermined directions, releasing molten material onto the surface to solidify. After finishing the first layer, the printer adds a second layer. This procedure of printing cross-sections is continued, layer-by-layer, until the entire object has been produced. This method is the most common because of its scalability and it is used to make a variety of 4DP products, such as bone samples for material behaviour testing in polymer laboratories, robot grippers, etc. [61]. FDM is an intriguing manufacturing method in the domain of tissue engineering due to its capacity to create porous polymer scaffolds [42].

### 3.2. 4DP Stimuli

In 4DP, the final product generated after the manufacturing changes its size, shape, color, etc., when stimulated or coming in contact with external media called stimuli. As a result, stimuli are required to initiate the transition in time, which is the fourth dimension of 4DP. The stimuli may therefore be water, heat, light, electrical currents, etc. The fourth dimension is the parameter that changes over time, such as size, shape, color, property, function, etc. The following are some examples of commonly used stimuli [59]

#### 3.2.1. Water or Solvent

It has been established that water, moisture, or liquid are crucial stimuli for accomplishing smart transformation. Temporally or spatially, liquid-responsive materials undergo a transformation in the form of surface expansion. Water-responsive hydrogel film transforms its shape, because of which it has varied applications. It has been reported that poly- (ethylene glycol) diacrylate hydrogel films exhibit reversible bending when exposed to moisture [31]. Similarly, multiple-layered poly-glycerol sebacate strips exhibited a similar reversible deformation when exposed to organic solvent vapor [32]. Using a polyethene glycol-based hydrogel, self-folding cylindrical scaffolds for cell encapsulation were made [62]. However, the liquid-responsive hydrogel has a delayed response time, a short life cycle and lower mechanical qualities as a result of expansion, swelling and potential degradation/hydrolysis owing to prolonged interaction with water. To address this, the swelling of the hydrogels must be designed to account for anisotropy. A group of researchers blended cellulose fibrils with hydrogen ink, which aligned primarily because of the generation of shear forces caused by the interaction of the print bed and hydrogen ink [63]. This alignment resulted in transverse swelling becoming four times that of the longitudinal swelling, enabling the programming of the 4D-printed structure [64]. In biomedical applications, liquid-responsive materials are primarily employed for cell encapsulation, controlled drug delivery, and reversible activation of smart valves.

#### 3.2.2. Temperature

Shape-memory polymers alter their shape when subjected to a dynamic mechanical force at a higher temperature. When such materials are subjected to a changing load, heated above the Tg and cooled, they deform into a transitory metastable shape. Additionally, when adequate transformation energy is employed during the application, the temporary shape deforms and then returns to the desired shape [41]. Consequently, such desirable viscoelastic and recovery properties of polymer have applications in bio-printed parts, such as self-conforming replacements for minor bone defect implants. These temperature-responsive bio-implant materials have been developed by a number of researchers, with approximately 98% form restoration [50]. For 4D printed bio-structure, when temperature stimuli are applied to a shape-changing material, the material’s shape and size change, and when the stimuli are removed the material returns to its initial shape [65,66].

In general, the biomedical efficacy of thermo-responsive polymeric materials is governed by their thermal characteristics, such as their Tg. In most cases, the components are pre-formed at a temperature that is higher than the Tg, which will cause the printed components to contract and become denser. These components are then cooled below their Tg to keep them small and compact for minimally invasive surgical procedures. When implanted into a body with a temperature greater than the Tg, these printed components regain their original, ideal form [67,68,69]. However, this approach is only applicable to substances whose Tg is below or equal to body temperature. Alteration or fabrication of biocompatible materials with a lower Tg or multi-responsiveness, where the material simultaneously responds to many stimuli, is required to broaden the base of such materials [70].

#### 3.2.3. Light

Light irradiation, curing and polymerization generated diverse shape and size transitions in polymeric substances [38]. Using the FDM printing technique, polyurethane-based material was manufactured and it was discovered that the size of the plate returned to its original cubic structure when illuminated with 87 mW/cm^2^ of light [71]. Light has also been utilized to achieve selective drug release from the capsule, for instance, capsule printing in a core-shell configuration, with an aqueous core loaded with the desired drug and the poly(lactic-co-glycolic) acid shell encapsulating plasmonic gold nanorods. By irradiating these capsules with a laser of a specified wavelength corresponding to the nanorods’ resonance wavelength, the capsule may be ruptured. Such rupturing might result in the sequential release of selected drugs, which is particularly significant for cancer therapy and the treatment of multiple-drug resistance [72].

The application of light as a stimulus has created a new opportunity for the development of bio-responsive medical equipment. However, more study is needed to address concerns regarding the possible toxicity of photo-activated materials, the production of heat during photothermal conversion, and the diminution of shape transformation due to the oxygen inhibiting action of the NOA65 composite [73]. Furthermore, when a photo polymerization method (such as SLA or DLP) is utilized to manufacture a light-responsive structure, photo-activated materials are restricted only to responding to specified wavelengths of light. To prevent undesirable conformation or structural transitions during printing, these specified wavelengths must not be within the wavelength range employed during the UV/laser printing procedure. If utilized as an internal implant, it is also important to examine the mode for triggering light stimulation. For instance, if an external light stimulus is delivered, then its penetration depth must be adequate and safe for substantial alteration to occur [74].

#### 3.2.4. pH

Localized acidification around cancerous or inflammatory regions and the variation in pH along the gastrointestinal tract have encouraged the adoption of pH-responsive materials to attain regulated anticancer drug delivery and organ-specific drug release, respectively. Due to the protonation of ionizable groups or degradation of acid-cleavable bonds [75,76,77], pH-responsive materials are capable of swelling, contracting, dissociating, or degrading in response to changes in external pH. When there is a shift in pH, materials that are pH-responsive undergo a globule-to-coil transition. This occurs when polymer chains either stretch into a coil shape owing to the electrostatic repulsion of charged functional groups, or form a globule structure when the charge of the functional groups is neutralized [78,79]. It is well known that, for drug release applications, a wide range of pH-sensitive synthetic polymers has been used.

However, 4DP was used to create the required systems for drug delivery with specialized designs and precise dimensions, both of which are impossible to achieve using conventional manufacturing processes. This was made possible through the utilization of 4DP technology. For instance, a hydrogel based on acrylic acid has been created and printed in order to produce tablets that are capable of causing rapid drug release in high pH conditions [80]. This tablet showed gastric resistance qualities as well as a drug release pattern that is pH sensitive which makes them a potentially useful approach for enteric drug delivery applications [81].

## 4. Advancement in 4DP

**Figure 2:**
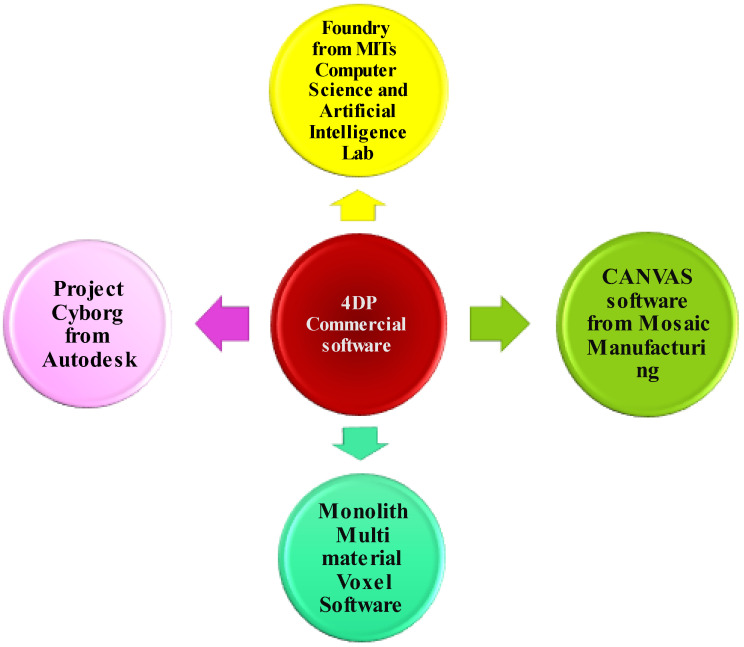
Some commercially available 4DP software: Foundry, Canvas, Monolith multi-material voxel and Project Cyborg. These software systems are used to make 4DP easier, efficient and cost-effective and are currently employed to control the shape-changing system in a well-ordered manner.

In recent years, a substantial effort has been made to build effective 4DP systems. The introduction of six different types of software solutions to assist the various stages of the 4DP process makes it easier and more efficient [7,22]. This can be listed as simulation, modelling, slicing, host/firmware, monitoring, and printing management software, respectively. A few commercial software packages are being used to structurally control shape-changing systems. Existing tools for self-assembly simulation and printing parameter optimization includes Foundry from MIT’s Computer Science and Artificial Intelligence Lab, CANVAS software from Mosaic Manufacturing, Project Cyborg from Autodesk, and Monolith multi-material voxel software. Furthermore, programming tools such as Origamizer and E-Origami Systems can assist in the creation of complex origami shapes by assigning nodes, paths, edges, polygons, vertices and creases to the structures [82]. The collective use of this software is essential for producing 4D printed products, as shown in Figure 2.

## 5. Advancement against 3DP

The use of 4DP technology may create objects whose dimensions and properties can change over time with variations in environmental factors, such as temperature and light changes. Some advantages of 4DP can be listed:

- Characteristics of the printed product: 4DP enables users to print objects utilising smart material, which has a variety of applications in health care, engineering and materials science.
- Resolution of printed objects: By employing 4D P technology, high resolution objects can be printed, which is difficult to achieve with the application of 3D printing.
- Alteration of object shape according to stimulus: The shape of a 4D-printed object changes over time in response to parameters such as light, temperature, pH, and so on.
- Innovative Technique: In this technology, the product is innovated during the design and development phases.
- Self-Assembly: This technique is likely to produce products with self-assembling properties due to the use of smart materials.
- Cost-effective: Reduced cost of manufacturing printed products as compared to 3D printing.

The literature categorizes 4DP technology into four primary printing techniques: stereolithography, fused deposition modelling, powder bed, and inkjet head 3DP. These technologies rely mostly on flexible materials with excellent mechanical and thermal qualities. However, the future relevance of this technology lies in the redefinition of manufacturing-related sectors that can complement existing manufacturing methods’ deficiencies. The main difference between 3DP and 4DP is that 3DP can generate static, non-temporal output (dimension) by employing various materials, such as powders, metals, thermoplastic polymers, UV-curable resins, etc., whereas, 4DP utilizes only materials that respond to changes in temperature, humidity, pressure,, etc., with the passage of time [83]. Table 2 depicts the evolution process of 4DP from 3DP. The table draws a comparison between 4DP and 3DP in regard to parameters such as built process, material, application, etc. The 4DP is more dynamic and flexible in comparison to 3DP. 4DP uses smart materials that are dynamic in nature and respond to stimuli, whereas 3D does not. Further, the scope of application of 4DP is wider as compared to 3DP.

## 6. Application of 4DP in Health Care

**Figure 3:**
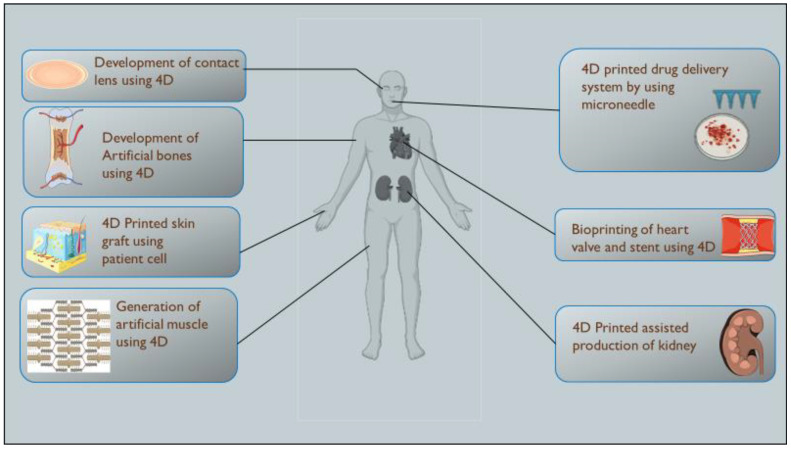
Application of 4D Printing in Healthcare which includes 4D printed organs, implants, tissue, etc.

**Figure 4:**
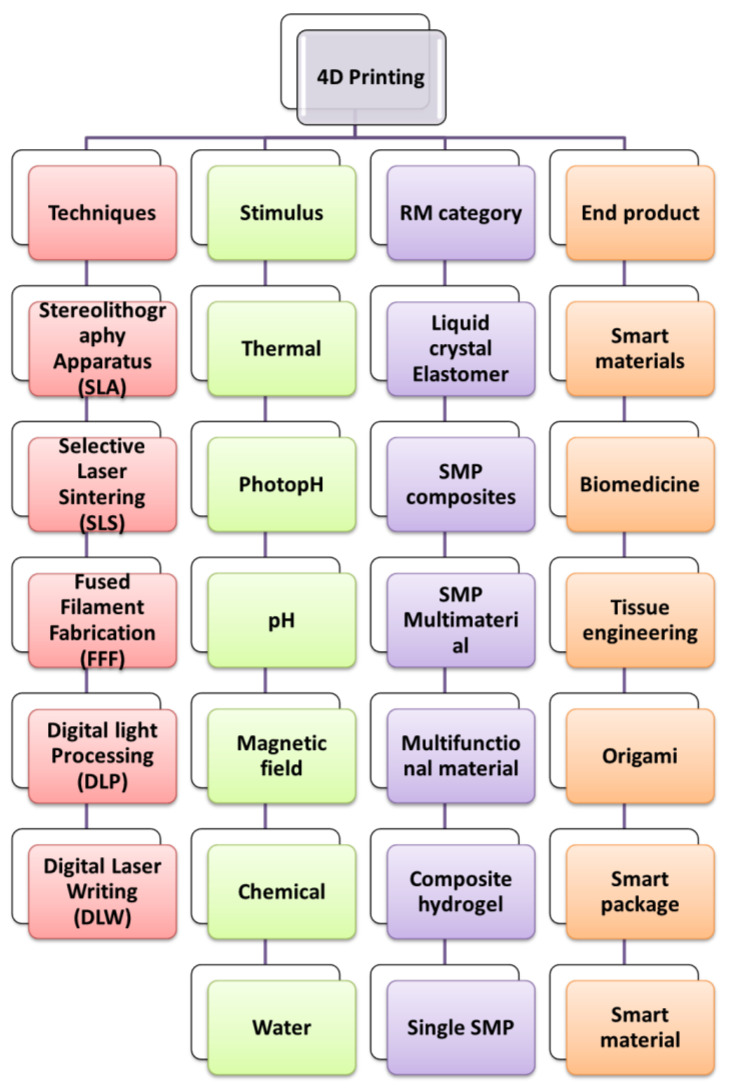
A brief description of 4D printing technology which includes the various techniques involved in the printing. The various materials used as raw materials with the common stimulus they act upon like pH, chemical, magnetic field and their useful end products such as biomedicine, tissue engineering, etc.

The 4DP offers a wide scope of applications in healthcare. It is used for grafts, biomedical devices such as smart stents, artificial tissue production, etc. It is also used in organ printing, such as heart, liver, etc. The smart devices manufactured using this technology are more biocompatible and adjustable to the body. 4D bioprinting has demonstrated the most recent technique for producing stimuli-responsive stents of comparable size. Various 4D bioprinting techniques and materials for stents have been developed. Upon implantation, stents would self-deform to the appropriate size and shape. Consequently, surgical invasions might be reduced. Tissue engineering and, more specifically, skin bioprinting provide a viable therapy for severe burns, surgical wounds and skin fragility illnesses. In comparison to skin transplants obtained from unaffected portions of a patient’s body, printed skin is believed to offer faster recovery, less discomfort and perhaps a more acceptable result. The time-dependent aspect of 4DP plays a vital role in the bioprinting of organs such as the heart, kidney, liver, etc. Another possible application of 4DP technology is drug delivery. It is possible to achieve localised drug release within the body by adjusting the transition temperature point of thermo-responsive materials nearer to physiological temperature and achieving a broad transition temperature range. For drug delivery, porous polymers are employed as drug carriers due to their low weight and larger area. Table 3 and Figure 3 depict several applications of 4DP in health care. Figure 4 gives a brief description of 4DP, includes the various techniques involved in this printing and the diverse items used as raw materials, with the common stimulus they act upon such as pH, thermal and magnetic field, and their useful end products such as biomedicines, tissue engineering etc.

## 7. Patents

The adoption of disruptive technology seldom comes without difficulties, just as with the introduction of personal computers and the internet. Although markets and industry have utilized 3D to its fullest potential, it was revolutionary for implants, pharmaceutical and other related industries. The technology started to catch on when high-quality 2D and 3D printers, respectively, became more reasonably priced at the end of the 2000s. By the year 2010, 4DP came into the market and assisted innumerable industries with its uniqueness and effective evolutionary research in a very short duration of time. It increases productivity, performance and perfection of product design. In the near future, 4DP may experience the same success as accomplished by 3DP. It will be definitively more effective, efficient and productive than 3DP. It is also critical to address important issues pertaining to 4D-printed pharmaceuticals and implant technologies/products, such as tort responsibility and intellectual property rights, in order to protect producers and end users. A collection of some noteworthy and recent healthcare research-related patents is listed in Table 4. The table tracks the various patents filed for 4DP. The table enlists patents dated from 1990 to 2017. The patents have been filed regarding shape memory polymers, additive manufacturing, bioactive medical devices, etc. Most of the research on 4DP has been carried out in the USA as major patents have been filed by researchers there. Few patents have been registered as global patents also.

**Figure 5:**
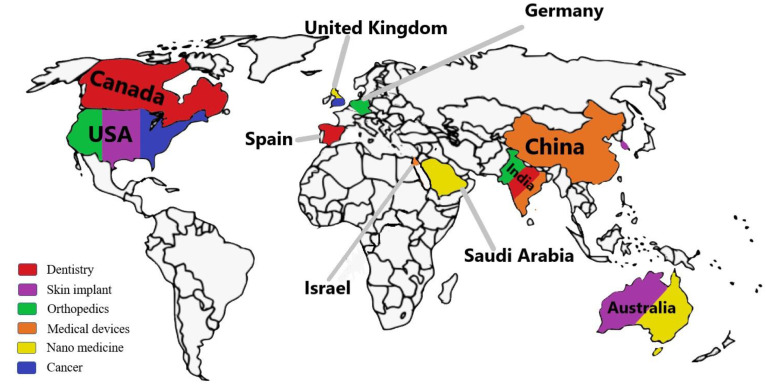
Depicts a world map on recent research publications that utilize the 4DP technique in various fields of healthcare such as nano medicine, medical devices, implants, etc; Publications from US and India cover wide range of healthcare-based research in comparison to other countries.

The first study on 4DP was published in 2013 (a year after the TED speech). Using the shape memory phenomenon, a printed sheet was transformed into a sophisticated structure by using the idea of printed active composites (PACs) (SME). In pharmaceuticals, medical implants and chemistry, many patents in 4DP have been registered and authors have listed some unique inventions in the above table [93]. For the control system used for a dot matrix printer developed by Iwamoto, Nagano et al. in 1990, comprising the creation of a printing device having a print head that is moved with a print medium in a print direction to implants, compatible with the surrounding area of the body where they are inserted mechanically, morphologically, and physiologically, the 4DP system has been invented and patents have been filed [94]. Additionally, Figure 5 depicts a world map on recent research publications that utilize the 4DP technique in various fields of healthcare such as nano medicine, medical devices, implants, etc. This shows that publications from the US and the India cover wide range of healthcare-based research in comparison to other countries.

## 8. Clinical Trials

The use of 4DP in the medical industry is promising. However, it is obligatory to assess the state of research and determine the potential applications for this novel collection of technologies. According to an assessment of the literature, 4DP is a more sophisticated version of 3DP. Here, changes in a 3D-printed product’s functioning, characteristics and shape are a function of time, with the capability of achieving self-assembly, self-repair and multifunctionality systems. As a result, 4DP can create dynamic structures, customizable shapes and objects with various functionalities. To accomplish this, 3DP technology employs smart materials and applies mathematical modelling in the construction of a structure. 4DP will undoubtedly benefit medical professionals, particularly in areas where 3DP technology is not yet available. 4DP assists in their production by layering intelligent material onto a 3D physical object utilizing computer-controlled CAD data. As a result, printed goods can take on a new dimension of alteration over time as they become more sensitive to factors such as temperature, humidity and time. This technology has the potential to significantly benefit the medical industry by providing better and more intelligent medical implants, instruments and devices. Researchers and physicians can exploit 4DP technology to provide the best patient care to those in need. The paragraph that follows provided a examples of inventions in the field of medical science and technology.

The 4DP can be utilized in cardiac MRI to measure pulmonary arterial pressure in patients with pulmonary hypertension. Some studies can be found on the identification of hepatic fibrosis using 4D-MRI, 4D-AC (4dimensions printing acute coronary syndrome); 4D-710 in adult patients with cystic fibrosis, cardiac and vascular evaluation using 4D-flow magnetic resonance (4DCARE); 4D-150 in patients with neovascular age-linked macular degeneration; prevention of aneurysm diameter enlargement after endovascular aortic repair of the abdominal aortic aneurysm through side branch embolization by means of preoperative 4D Flow MRI analysis; 4D-flow cardiac MRI to evaluate pulmonary arterial pressure in pulmonary hypertension; 4D CBCT and Intra fractional imaging for the determination of the most representative 4D simulation planning technique for lung SBRT technique; identification of novel markers of atrial myopathy in patients with embolic stroke of undetermined source (ESUS) from MRI 4D Data,, etc., are some of the majorly studied topics. Detailed information on clinical trials is described in Table 5. The table contains information about clinical trials that have been conducted recently in the years 2021 and 2022. Most of these trials are for the prevention/treatment of cardiovascular ailments. This data generates proof for supporting applications of 4DP in the area of medical science.

## 9. Future Perspective

Current advancements in 4DP offer a promising future in which this technology can be exploited in a variety of industrial and manufacturing areas. To create a prosperous future for 4DP, it is necessary to develop novel, highly customizable materials that can respond to many external stimuli in order to undergo their respective shape transformations. Nevertheless, it is critical to develop new 4DP software for various 4DP techniques while taking into account factors such as the base smart material, geometrical and structural requirements of the product, printing technique and the shape-changing mechanism of the 4DP techniques during software development, among others. Furthermore, the development of 4DP techniques that can adapt to different materials rather than a single material is essential for its success. 4DP has enormous potential in many subfields of biomedical engineering. However, it will take a significant amount of effort to develop faster, more cost-effective bio-printing techniques.

It is essential to produce biocompatible smart materials which should not be constrained in their compatibility and have the capacity to provide complete functioning with diverse tissue and stimuli systems [95]. The next generation of smart biomedical tools must experience complex motions in order to function more successfully within the human body. Further, prompt research should be carried out to develop novel biopolymer and multifunctional materials that should be adapted for biomedical device fabrication and be capable of performing numerous functions individually [96]. Additionally, it is crucial to develop completely automated low-cost, and economically viable bioprinters with an excellent resolution that will support the expansion of this technique [97,98].

**Figure 6:**
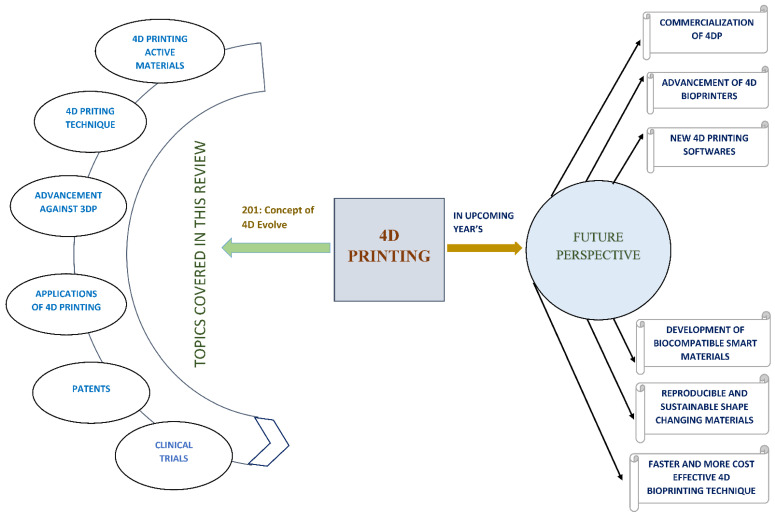
Summary of future perspective of 4DP with a focus on the methods that can be adopted to increase the commercialization of 4DP. Moreover, the figure outline the issues addressed in this review.

Moreover, it requires the development of new smart materials for unique shape programming methodologies and the generation of more complicated structures to broaden the list of existing smart materials used in 4DP via extensive research. Another major area of 4DP study is the degradability of structures. It is well-known that changeable-shape structures are susceptible to degeneration and hence demand extensive study to make the shape-changing process reproducible and more sustainable over time. Figure 6 tries to demonstrate a concise summary of future perspectives while highlighting the topic covered in this review.

In addition to the functions listed above, 4D printed objects have potential applications in human body systems such as artificial muscles, wearable sensors and implanted biomedical microdevices, among others. When printed enzymes are used, this technique can have an impact on tissue engineering concepts such as vascularization and tissue formation. Future devices that cover wounds and prevent further corrosion by stomach acid may include self-deformed microcapsules that can create with 4D technology. This technique has enormous potential for use in the printing of personalized contact lenses and personal hygiene items. Other practical uses include the construction of by-products consisting of shape-changing photovoltaic solar cells and deployable aeronautical structures, such as morphing aircraft wings. Fascinatingly, 4DP may also be used to create dynamic jewelry, such as temperature-sensitive rings, and footwear accessories, such as shoes with smart heels. The commercialization of 4DP technology remains in its initial phases but this developing technology will contribute to a more sustainable environment by reducing the wastage of natural resources, hence, decreasing long-term costs and providing sustainable alternatives to existing items. Furthermore, the 4DP market is expected to see a surge in the release of new eco-friendly products and collaborations across the value chain, giving rise to resource-efficient and sustainable consumption.

## 10. Conclusions

Since its inception, 4DP has grown significantly and spread its influence across different industrial divisions such as aerospace, medical and defense industries. 4DP typically combines 3DP technology with smart material to produce a mechanism for changing shape across time. It offers superior adaptability and versatility because a single structure may serve multiple functions. There is no doubt that the future of 4D printed materials is optimistic and bright. It has the potential to eliminate mechanical systems’ inherent flaws while increasing output. By utilizing 4D-printed smart materials, novel, degree-of-freedom-unrestricted systems could be created. However, these innovative systems are created by replacing conventional mechanical components such as motors and gears with smart materials. Further, advanced smart devices can be improved by incorporating electronics into smart structures in a novel and more efficient way. Moreover, the advancement of biocompatible smart material systems has the potential to revolutionize the medical industry and improve treatment efficacy. Lastly, it is difficult to picture a future in which 4DP has no significant influence on the industrial sector.

## Figures and Tables

**Table 1:** Raw materials used in 4D Printing with their application and properties. The raw materials include SMPs, SMAs and Magnetic Nano Particles having specific stimuli like temperature, pH, etc., with their characteristic properties. The applications of some are also mentioned, such as in drug delivery systems, biomedical devices and smart muscle. SMPs may be used as copolymers. SMAs generally respond to temperature.

S.NO	Raw Material (Polymer/Alloy)	Stimuli	Application	Additional Properties
1.	Nitinol Shape memory alloy, composition-50%- Nickel and 50%Titanium	Temperature	Wires, biomedical devices	Biocompatible, super elastic, ↑ corrosion resistant with non-ferromagnetic characteristics
2.	Ni-Mn-Ga based alloy (SMA) (Ferromagnetic shape memory alloy)	Temperature & magnetic field	-	Reverse shape transformation ability
3.	Fe based Shape Memory Alloy (SMA)	Temperature	-	Cost-effective & ↑ pseudo elastic strain
4.	Copper based Shape Memory Alloy (SMA)	Temperature	-	Cost-effective having poor ductility & poor martensitic stabilization
5.	Acrylic Acid IUPAC Name-Propenoic acid CAS No-79-10-7	pH	Drug delivery system	The mechanism of their response to pH is by crosslinking and dissociation on interaction with ions
6.	Polycaprolactone (PCL) CAS No-24980-41-4 IUPAC name- (1,7)-Polyoxepan-2-one	Temperature	Drug delivery system, splints, personalized stent	Biocompatible having glass transition temperature 60 °C and melting point-56–65 °C & used with other compounds such as methyl-acrylate
7.	PASA—Poly (aspartic acid) IUPAC Name-2-aminobutanedioic acid CAS No 25608-40-6	pH	Tissue engineering application	Biocompatible & biodegradable
8.	PNIPA, PNIPAM, PNIPAAm, NIPA, PNIPAA or PNIPAm- Poly(N-isopropylacrylamide) CAS No-25189-55-3	Temperature	Artificial muscle, biomedical devices	Lower Critical Solution Temperature (LCST) (32 °C) above which swelling occurs, forms copolymer with nonactive polymer-Phema(poly[2-hydroxylethyl methacrylate]) because at critical temperature the polymer undergoes ‘coil to globule ‘ transition
9.	Polyurethane hydrogel (PU) and copolymers CAS No- 9009-54-5	Mechanical force & temperature	Drug delivery system, biomedical device	Biodegradable having a glass transition temperature 121 °C, Polyurethane composites are made by using carboxy methyl cellulose (CMC)/SiO_2_, viscosity alters in pure PU and composite PU
10.	Perylene bismide-functionalized hyperbranched polyethyleneimine (PBI-HPEI) and graphene oxide-PNIAm (GO-PNIAm) hydrogel Graphene oxide CAS No- 1034343-98-0	Temperature & pH	-	-
11.	Poly(Vinyl alcohol) IUPAC Name- Ethanol CAS NO-9002-89-5	Moisture	Gastro-retentive & intravesical drug delivery system	Biodegradable having water-responsive shape memory behaviour & hot-processing capability
12.	Magnetic Nanoparticles (MNP) (Fe, Co, Ni or their oxides)	Magnetic field	-	-
13.	Poly (ethylene-glycol) diacrylate (PEGDA) CAS No-26570-48-9	Moisture	-	Bio-restorable
14.	Polylactic Acid (PLA) CAS No-26100-51-6	Temperature and magnetic field	Occlusion devise, bone scaffolds	Good biocompatibility & controlled biodegradability, used with hydroxyapatite nanoparticles & shows responsiveness to magnetic field on the addition of Fe_3_O_4_
15.	Polydopamine (PDA) IUPAC name-Poly(3,4-dihydroxyphenylethylamine) CAS N0-86389-83-5	Photothermal	Biomedical engineering	Dopamine-derived polymer shows biocompatibility

Note ↑: Increase.

**Table 2:** Evolution of 4D printing from 3D printing. 4D P has evolved from 3D printing. The table draws a comparative comparison between 4D and 3D for various parameters such as built process, material, application, etc. The 4DP is more dynamic and flexible in comparison to 3D. 4D uses smart materials that are dynamic that respond to stimuli whereas 3D does not. The scope of application of 4D is wider as compared to 3D.

S.No	Parameters	3D Printing	4D Printing
1.	Built Process	3DP repeats layer by layer composition of 2D from base to top	4DP is a step forward from 3DP
2.	Materials	Thermoplastics, metals, ceramics, biomaterials/nanomaterials	Material that is smart, multi-material and self-assembling to build an object that changes shape after it is manufactured. Nevertheless, new materials must be developed to meet the needs of the applications
3.	Structure Flexibility	Non-flexible, characterized by the rigid structure	Flexible, final structures are versatile
4.	Object shape Configuration	The shape of the object changes	The shape of the object is changed concerning time, etc.
5.	Programming of Smart materials	Do not utilize any programmable & smart material	Use programmable & smart materials that can provide a variety of benefits
6.	Printer used	3D printer	Smart/multi-material 4D printer
7.	Product State	Static products	Smart, dynamic Products
8.	Applications	Its applications include healthcare, dentistry, engineering, automobiles, toys, aerospace, jewelry and defence, etc.	3DP’s dynamically changing configuration for all applications

**Table 3:** Application of 4D printing in health care. This includes 4D Printing of various vital organs such as kidney, bones and heart valve. For manufacturing of dosage forms such as tablets, using 4DP will scale up their production.

S.NO.	Medical Application	Description	References
1.	Stents	4DP potentially produces stents that can expand and attain the desired shape with the aid of the patient’s body heat. This innovative technique saves a patient’s life during a complex surgery by acting swiftly. A shape varies concerning time and temperature. Stents comprised of shape-memory material and a support framework have been developed as a non-invasive treatment option for blocked heart vessels.	[38,84]
2.	Organ Printing	This new technique can be used to manufacture complicated 3D organs. Used to print organs by using a patient’s cells and hence save a life. It could be used to treat organ scarcity.	[85]
3.	Printing of Cornea implants	Interpenetrating polymer networks (IPN) scaffolds were employed to create artificial cornea implants. Utilizing IPNs permits the construction of three-dimensional scaffolds with great strength and water retention capacity. These characteristics along with their well-defined, very porous structure result in the creation of devices that are highly biocompatible.	[86]
4.	Implants for Dyspnea (breathing Problem)	Babies afflicted with dyspnea (breathing problems) are saved by 4D printing technology. It rapidly generates a medical implant that can change form as newborns grow, allowing them to continue breathing.	[87]
5.	Tissue Engineering	The fabrication of SMPs into ultrafine nanoscale fibers yields scaffolds for tissue engineering. These nanoscale structures combine the advantageous properties of nanofibers & SMPs to create a system that is readily biodegradable & efficient in bone & tissue repair & regeneration. Nano-bio-composites of natural polymers are utilized to construct scaffolds for generating bone tissue.	[88,89]
6.	Printing of Heart, Kidney and Liver	Future 4DP can manufacture the heart, kidneys and liver utilizing smart materials. The capability to manufacture these components with a high degree of adaptability and exact genetic match up.	[90,91]
7.	Skin Regeneration	It offers significant potential for printing skin regeneration in the patient’s original color. Also beneficial for patients with burns, as it readily integrates into the patient’s body and develops like the original. Multiple layers of keratinocytes, collagen and fibroblast of the patient’s skin are utilized to regenerate the skin	[43,92]

**Table 4:** List of Patents for 4D Printing Globally.

S.No	Inventor	Title	Date of Filing	Description	Patent Number
01	Jennifer A. Lewis, Amelia Sydney Gladman	Method of 4d printing a hydrogel composite structure	30 November 2015	A technique for printing a hydrogel composite structure in 4D	US20170151733A1
02	Peter A. Feinstein	Hybrid smart assembling 4D material	2 February 2015	The substance consists of a composite comprising shape memory and non-shape memory components, as well as a trigger source connected to the form memory component	US9427941B2
03	Skylar J.E. Tibbits, Daniel Dikovsky, Shai Hirsch	Object Of Additive Manufacture with Encoded Predicted Shape Change and Method Of Manufacturing Same	25 February 2014	An object consisting of an additive manufacturing material that responds to an external stimulus and is configured to cause a predicted transformation of the object from a 1st manufactured shape to a 2nd manufactured shape in response to the external stimulus	US20150158244A1
04	Jeffery Adam WEISMAN, Connor NICHOLSON, David Mills	Methods and devices for three-dimensional printing or additive manufacturing of bioactive medical devices	10 August 2015	A technique for manufacturing a bioactive implant	WO2016025388A1
05	Yu Ying Clarrisa CHOONG, Saeed MALEKSAEEDI, Hengky ENG, Pei-Chen Su	Shape memory polymer, formulation for, method of forming and device including the same	20 April 2017	A method of creating the shape memory polymer, & a device containing the polymer such as a suture, stent/a dental aligner	WO2017188896A1
06	Jennifer Nicole Rodriguez, Eric B. Duoss, James Lewicki, Christopher SPADACCINI, Thomas G. Wilson, Cheng Zhu	Additively manufacturing bio-based conductive shape memory polymer macrostructure parts with highly ordered microstructures	25 October 2016	An additive manufacturing apparatus consists of a print head for additive manufacturing and a nozzle that accepts a bio-based shape memory polymer material	WO2017078987A1
07	Kazuhiko Sato, Kenichiro Arai, Hiroshi Narita	Ink pump selective driver and ink jet printer incorporating the same		A pump driver for selectively driving a number of pumps, which includes a drive source and a sun gear that is rotated by the drive source. a planetary gear that is meshing with the sun gear; and a planetary carrier that rotatably supports the planetary gear as it revolves around the sun gear	US6761438B2
09	Naohisa Iwamoto, Tadashi Nagano, Syuhji Sugita, Shin Yamas	Control system for a dot matrix printer	10 October 1990	A printing apparatus with a printhead that moves in a print direction relative to a print medium	US5171093A
10	Jeffery Adam WEISMAN, Connor NICHOLSON, David Mills	Methods and devices for three-dimensional printing or additive manufacturing of bioactive medical devices	10 August 2015	A technique for creating a bioactive implant	WO2016025388A1
11	Randy-David BurceGrishaber, Daniel Olsen	AAA model for fatigue testing	18 January 2006	The technique of creating a 3D model of the test apparatus with a CAD program & applying said method using a flexible material to the 3D model to create the flexible test apparatus	US20070168066A1
12.	Jan Zwijsen	Detection of type of dye donor element in a thermal printing system	12 April 1994	A thermal printing system equipped with a minimum of 3 light sources & 3 photodetectors positioned opposite each other for detecting the type of dye donor element allows for the distinction of a dye donor element for color printing & a dye donor element for black & white printing, as well as the detection of additional variants	US6080993A
13	Fabien Beckers, Albert HSIAO, John AXERIO-CILIES, Torin Arni TAERUM, Daniel Marc Raymond BEAUCHAMP	Apparatus, methods and articles for four dimensional (4d) flow magnetic resonance imaging	16 January 2015	An asynchronous command & imaging pipeline enables remote image processing & analysis in a timely & secure manner, even with complex/large 4-D flow MRI data sets	WO2015109254A3
14.	Ivan Stangel, Walter Zimbeck	Production of dental restorations and other custom objects by free-form fabrication methods and systems therefor	20 December 2002	A process for producing a dental restoration/dental restorations	US20030222366A1
15.	Razvan IoanIonasec, Ingmar Voigt, Viorel Mihalef, SasaGrbic, Dime Vitanovski, Yang Wang, Yefeng Zheng, Bogdan Georgescu, DorinComaniciu, Puneet Sharma, Tommaso Mansi	Method and System for Comprehensive Patient-Specific Modeling of the Heart	20 April 2011	A method & system for patient-specific modelling of the whole heart anatomy, dynamics, hemodynamics & fluid-structure interaction from 4D medical image data	US20120022843A1
16	Razvan IoanIonasec, Puneet Sharma, Bogdan Georgescu, Andrey Torzhkov, Fabian Moerchen, Gayle M. Wittenberg, Dmitriy Fradkin, DorinComaniciu	Method and system for multi-component heart and aorta modeling for decision support in cardiac disease	30 April 2010	A technique and system for producing a patient-specific anatomical heart model	US8527251B2
17	David Opie, TranquocThebao Nguyen, AtakanPeker	Medical implants	19 August 2003	A bulk-solidifying amorphous alloy medical implant and methods for making such implants, in which the medical implants are biologically, mechanically, and morphologically compatible with the body	US9795712B2
18	Joe Giglio, Fred Schiegel	Kiosk with body fat analyzer	5 April 2001	An apparatus for evaluating a user’s physical condition based on personal data supplied by the user and measurements taken by the device, with output displayed on a screen and/or printed	US20040044560A1
19	PalmiEinarsson	Ventilated prosthesis system	21 March 2017	A ventilated shell and a substantially compliant, ventilated spacer element that define a first surface with a frictional feature comprise a prosthesis system	US7488349B2
20	Angele Sjong, William Brenden Carlson, Michael Keoni MANION	Packaging materials and methods for their preparation and use	23 July 2013	Food packaging and methods of making and using it; it is an alteration to prevent content deterioration	WO2015012803A1
21	Michael Frenkel, Alexander Katsevich, Igor Frenkel	Systems, apparatus and methods for collecting and storing raw scan data and software for performing data processing, image reconstruction and interpretation	12 March 2013	Apparatus, systems and methods for collecting, storing, processing, reconstructing and interpreting raw scan data from a medical diagnostic imaging scan	US9235889B1

**Table 5:** Details of global clinical trials that employ 4D Printing Technology. The table contains information about clinical trials conducted recently in the years 2021 and 2022. The majority of these trials are for the prevention/treatment of Cardio-Vascular Diseases.

S.NO.	Trial ID	Public Title	Date Registration	Intervention	Sponsor(s)	The Phase of Study (Status)	Location
01.	NCT05385237	Identification of Hepatic Fibrosis Using 4D-MRI	17 May 2022	Diagnostic Test: 4D-MRI	University Hospital, Basel, Switzerland	Recruiting	Switzerland
02.	KCT0006992	4D-ACS study	10 February 2022	Drug: This clinical trial was designed as a prospective, multicenter, randomized, comparative study.	Gachon University Gil Medical Center	Recruiting	Korea, Republic of
03.	NCT05248230	4D-710 in Adult Patients With Cystic Fibrosis	10 February 2022	Interventional	4D Molecular Therapeutics	Recruiting	United States
04.	ACTRN12622000047796	Cardiac and vascular Evaluation using 4D-flow magnetic resonance (4DCARE)	17 January 2022	Patients referred by their doctor for Cardiovascular magnetic resonance imaging are invited to participate in the study.	Imaging and Phenotyping Laboratory	Recruiting	Australia
05.	NCT05197270	4D-150 in Patients with Neovascular (Wet) Age-Related Macular Degeneration	5 January 2022	Interventional	4D Molecular Therapeutics	Recruiting	United States
06	NCT05103189	4D-flow Cardiac MRI to Assess Pulmonary Arterial Pressure in Pulmonary Hypertension	21 October 2021	Other: 4D-flow sequence	Centre Hospitalier Universitaire, Amiens	Recruiting	France
07	JPRN-jRCT1042210068	A Prospective Study of Prevention of Aneurysm Diameter Enlargement after Endovascular Aortic Repair of Abdominal Aortic Aneurysm by Side Branch Embolization Using Preoperative 4D Flow MRI Analysis	13 September 2021	Side branch embolization will be performed in cases where the mass side branch blood flow is above the cutoff value on the preoperative 4D Flow MRI examination, according to the method specified in the study plan.	Sano Masaki	Recruiting	Japan
08	JPRN-UMIN000044386	Analysis of respiratory dynamics using 4D-CT during mechanical ventilation	1 June 2021	N/A	Jichi Medical University School of Medicine	Recruiting	Japan
09	ChiCTR2100046156	Prospective clinical study of PET-CT/4D-PCMR combined with IVUS multimodal imaging for risk stratification and predictive analysis of acute aortic intermural hematoma	6 May 2021	case series: PET/CT, PCMRI and IVUS scan	Department of Vascular Surgery, Zhongshan Hospital, Fudan University	Recruiting	China
10	NCT04867954	Development of 4D Flow MRI for Risk Stratification of Variceal Bleeding in Cirrhosis	27 April 2021	N/A	University of Wisconsin, Madison	Recruiting	United States
11	ChiCTR2100045687	Evaluation of ejection fraction based on four-dimensional automatic volume quantification and spot tracking technology to preserve left ventricular function in patients with heart failure	23 April 2021	Gold Standard: Brain natriuretic peptide, magnetic resonance, nuclide	People’s Hospital of Shapingba District, Chongqing	Recruiting	China
12	ChiCTR2100042953	Research on Delayed Cerebral Ischemia after Aneurysmal Subarachnoid Hemorrhage based on 4D-flow MRI	1 February 2021	Interventional group: Intervention.; Clipping group: Clipping; Conservative Group: Conservative treatment	Beijing Tiantan Hospital, Capital Medical University	Recruiting	China
13	NCT04735585	Kinematic Assessment of Human Peripheral Joints by Dynamic CT	21 January 2021	Procedure: Physiotherapy or Surgery	UniversitairZiekenhuis Brussel	Recruiting	Belgium
14	NCT04717843	Identification of New Markers of Atrial Myopathy in Patients with Embolic Stroke of Undetermined Source (ESUS) From MRI 4D Data	15 January 2021	Other: 4D Flow MRI; Other: Medical consultation with 12 leads ECG; Other: Holter ECG; Other: Trans thoracic echocardiography; Biological: Blood sample; Other: Standard MRI	Hospices Civils de Lyon	Recruiting	France
15	NCT04717804	4D CBCT and Intra-fractional Imaging for the Determination of the Most Representative 4D Simulation Planning Technique for Lung SBRT Technique Patients	11 January 2021	Other: AIP CT; Other: FB (Free-Breathing) CT	The University of Texas Health Science Center at San Antonio	Recruiting	United States

## Data Availability

Not applicable.
